# Neighborhood Characteristics Associated with Running in Metro Vancouver: A Preliminary Analysis

**DOI:** 10.3390/ijerph192114328

**Published:** 2022-11-02

**Authors:** Stella R. Harden, Nadine Schuurman, Peter Keller, Scott A. Lear

**Affiliations:** 1 Faculty of Environment, Simon Fraser University, 8888 University Drive, Burnaby, BC V5A 1S6, Canada; 2 Faculty of Health Sciences, Simon Fraser University, 8888 University Drive, Burnaby, BC V5A 1S6, Canada

**Keywords:** running, environmental preferences, built environments, crowdsourced data, Strava, GIS, public health

## Abstract

Running can improve physical health and psychological wellbeing. However, the characteristics of conducive running environments are relatively unknown. This study determines neighborhood factors that attract running and explores how age and gender mediate built environment preferences. Spatial patterns of runners in Metro Vancouver were identified using crowdsourced fitness data from Strava, a popular application for tracking physical activities. The influence of socio-economic status (SES), green and/or blue space, and urbanicity on route popularity was assessed using a Generalized Linear Model (GLM). The influence of these neighborhood variables was also calculated for runners by age and gender. The results show high neighborhood SES, the presence of green and/or blue space, and high population density are associated with increased running activities in all age and gender groups. This study contributes a novel approach to understanding conducive running environments by demonstrating the utility of crowdsourced data in combination with data about urban environments. The patterns of this large group of runners can be used to inform planning for cities that promote running, as well as seek to encourage equal participation among different ages and genders.

## 1. Introduction

In the past two decades, running has grown in popularity in cities of high-income countries [1,2,3,4,5,6] in part because it is more accessible than other physical activities and does not require specialized settings and equipment. Runners have diverse backgrounds and abilities, with some running competitively and others for leisure. Studies have identified the importance of increasing the accessibility of running by understanding the varying motivations and preferences within the community [3,4,7]. However, the environmental characteristics preferred by runners of different ages and genders is seldom discussed. Further, the built environment impacts running experience but the exact environmental features that influence running are unclear [8,9]. It is important that this relationship be identified as running has a multitude of benefits for physical and mental well-being. Running regularly is associated with reduced risk of cardiovascular disease, type 2 diabetes, and obesity [10,11,12,13]. At the same time that recreational running is increasing [6,14], so is sedentary behavior [15,16,17,18], which has the potential to undo benefits from running. Therefore, health initiatives have identified recreational running as an effective means to promote active populations and lower risks associated with physical inactivity [14,19,20,21].

Awareness of the environment as a direct contributor to the running experience, rather than as a backdrop, could improve public health initiatives and infrastructure planning [3,22]. For example, running near green spaces is associated with additional benefits to cardiovascular and mental health [10,23,24,25,26]. A growing body of literature highlights similar health benefits from blue spaces, which are water bodies such as rivers, lakes and the oceans [27,28,29,30]. It is no surprise then that studies report that runners prefer greener and bluer routes [27,28,31]. A number of surveys found runners prefer routes that are quiet, close to green space, and have comfortable surfaces [1,3,32,33]. In addition to these surveys, a rough index of conducive running environments was developed based on findings in walkability literature; however, the index was limited by the lack of research on runner-friendly environments [9]. While these studies begin to understand a desirable running environment, they lack real-world spatial data to compare identified preferences with the realities of running. Crowdsourced data offer opportunity to supplement survey findings reporting perceptions of ideal running spaces by providing direct insight into the correlation between runners and the running environment. 

Additional spatial characteristics associated with physical activity include socio-economic status (SES) and urbanicity. People living in neighborhoods with low SES are less likely to be physically active, due to less favorable built environments that discourage activities such as running [34,35,36]. Similarly, moderate urbanicity has been found to increase physical activity levels due to better street connectivity and infrastructure; however, highly urban centers have tended to have greater levels of physical inactivity [37,38,39]. While running is a component of physical activity, we cannot assume the determinants for one activity will be the same for another, or to the same degree.

Crowdsourced fitness apps have the potential to inform urban planning and infrastructure improvements. They are already being used to plan for cycling and may hold similar promise for running [40,41,42,43,44,45]. Strava, a popular fitness tracking app for runners and cyclists, links information such as pace, elevation gain, calorie deficit, and average heart rate with traversed routes. Additionally, the app has a social component where users can share their progress with followers. The spatial granularity and high volume of data provided by Strava Metro, the app’s data platform, opens new avenues for understanding runner behavior [40,46]. Research using Strava for cyclists is increasing [47] and has identified that route popularity is correlated with green space, urbanicity, and SES [45,48,49]. It remains unknown whether runners have the same preferences and needs as cyclists. Runners tend to spend more time in spaces than cyclists during a trip since their average speed is lower. Running thus relies more heavily on conducive environments for a better experience, indicating neighborhood settings may have a greater impact on runner choices than cyclist choices. This preliminary study contributes additional insight into runners’ environmental preferences and illustrates the potential of Strava data to assess the relationship between neighborhood characteristics and route popularity among Strava runners.

## 2. Materials and Methods

### 2.1. Study Area

**Figure 1:**
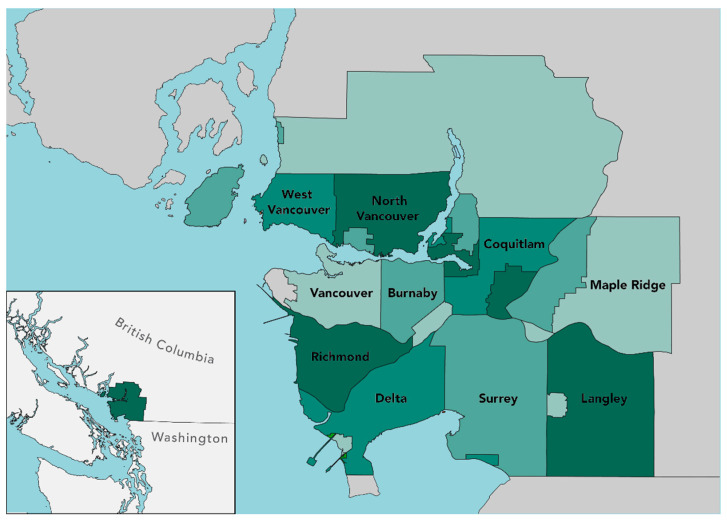
Map of study area in the context of the Pacific Northwest. The Tsawwassen First Nation Treaty, 21 municipalities, and one Electoral Area form the region known as Metro Vancouver.

The study area is Metro Vancouver, British Columbia, which is composed of 21 municipalities, the Tsawwassen First Nation Treaty, and one Electoral Area (Figure 1). Metro Vancouver had approximately 2,642,825 inhabitants in 2021 with key employment sectors in retail trade; healthcare and social assistance; and professional, scientific and technical services [50]. The coastal metropolitan area is in the Pacific Northwest at the confluence of the Fraser River delta and the Salish Sea. It has a moderate oceanic/semi-Mediterranean climate. Metro Vancouver has varying degrees of urbanicity which aids in understanding Strava running behavior in both urban and rural environments. Outdoor recreation spaces and protected natural areas accounted for 33.1% of the area in 2016.

### 2.2. Strava Data

Runner data for Metro Vancouver from all of 2019 was purchased from Strava Metro. Strava Metro anonymized and aggregated user activities in a spatial file made up of line segments representing routes, each divided into unique features by their intersections with one another. Segments contain count information for Strava users and running activities using bins of five to protect individual privacy. This binning method used by Strava results in segments with fewer than three recorded activities or users being counted as zero. Our study analyzed a total of 242,265 segments. In addition to overall user and activity counts, Strava categorized users by gender (man, woman, unspecified) and age (13 to 19, 20 to 34, 35 to 54, 55 to 64, 65 and older).

### 2.3. Neighborhood Variables

Green/blue space, SES, and urbanicity were included as categorical explanatory variables in the analysis due to their potential relationship with running based on the studies above (Table 1). To determine green/blue space, land use data from 2016 was collected from Metro Vancouver’s open access portal http://www.metrovancouver.org/data (accessed on 10 May 2022). A standardized definition of green space is not often mechanized by researchers. Generally green space is defined as a vegetated area associated with natural features, such as a park or trail [51]. In our study, green space consisted of open recreation spaces (i.e., playing fields, camping parks, and walking paths) and protected natural areas (i.e., parks, ecological reserves, and forests) as these areas primarily include natural features. Blue space was defined as visible waterbodies such as rivers/canals and coast, lakes and ponds. Segment contiguity with green/blue space was categorized as ‘absent’ or ‘present’, meaning a segment was within 15 m of a green and/or blue space. A 15 m buffer was selected due to the requirement for spaces to be within close proximity to a route, and many studies have found larger buffers in green space studies accurately predict health outcomes [52,53].

Deprivation indices, a composite measure of SES, have been developed to understand the impact of neighborhood characteristics on community wellbeing [54,55,56,57]. The Vancouver Area Neighbourhood Deprivation Index (VANDIX) measures SES disparities to understand variations in population health [57,58]. VANDIX, originally constructed as a proxy for public health status, is an SES metric specific to urban areas in British Columbia [54]. Prior studies have used VANDIX to analyze different health outcomes [59,60,61]; however, this study uses the metric to understand the influence of neighborhood SES on running patterns. Deprivation is calculated through a combination of seven weighted census variables, listed in order of importance: proportion without high school completion; proportion without university completion; unemployment rate; proportion of single-parent families; average income; proportion of homeowners; and employment ratio [58]. Scores were calculated for the 3461 dissemination areas (DAs) within the study area [62,63] and stratified into quintiles of roughly similar DA counts. Quintiles were selected to compare low, moderately low, moderately high, and high relative SES with a reference of moderate neighborhood SES.

Last, urbanicity was measured through the population density of DAs and stratified into quintiles of similar size representing low, moderately low, moderate, moderately high, and high urbanicity. The social elements of urbanization are better captured using additional measures such as proximity to education facilities, healthcare, paved roads, or transportation density [64]. For the sake of our analysis, using population density as a simple proxy for urbanicity was sufficient as population distribution ultimately reflected levels of urbanization in Metro Vancouver. Urbanicity, often measured by population density, is associated with amenities and infrastructure that characterize an urbanized environment and impact exercise behaviors [38,65].

### 2.4. Statistical Analysis

Statistical analyses were conducted using R software (V.4.2.1) [66]. A Generalized Linear Model (GLM) determined the main effects of SES, urbanicity, and green/blue space on route popularity, measured by the amount of activities along a segment. An analysis of deviance led us to select a negative binomial distribution as this was ultimately the best fit for the data. Variance Inflation Factors measured collinearity between the explanatory variables, and values were less than two indicating insignificant multicollinearity between the variables [42,67]. Subsequent regressions were conducted using separate models for gender and age categories to determine the relationship between neighborhood characteristics and runner volume within respective groups. The results of the GLM analyses are presented as incidence rate ratios (IRR) with significance at *p*-value < 0.001. Incidence Rate Ratios (IRR) were used to interpret the influence of each variable on Strava running activities. IRR coefficients explain the expected change of the dependent variable for each unit of the predictor variable [48,68]. 

## 3. Results

### 3.1. Descriptive Statistics

**Figure 2:**
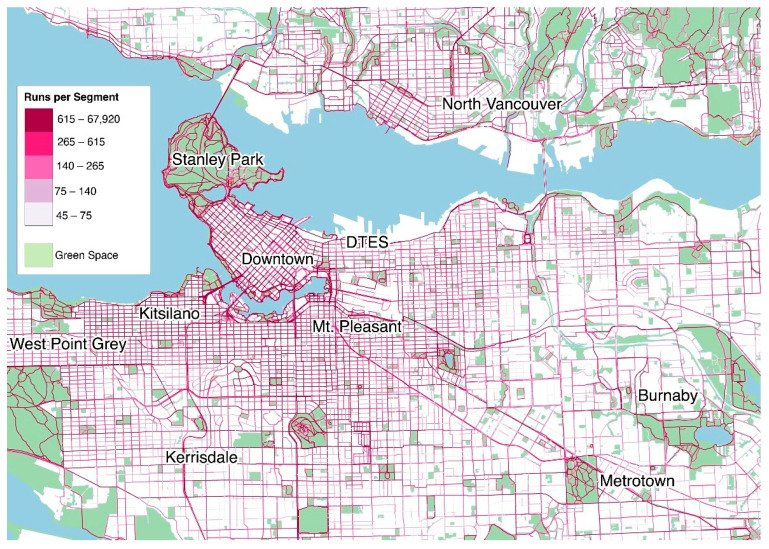
Distribution of Strava running activities in of Metro Vancouver. The majority of runs are observed in Downtown Vancouver and surrounding areas.

**Figure A1:**
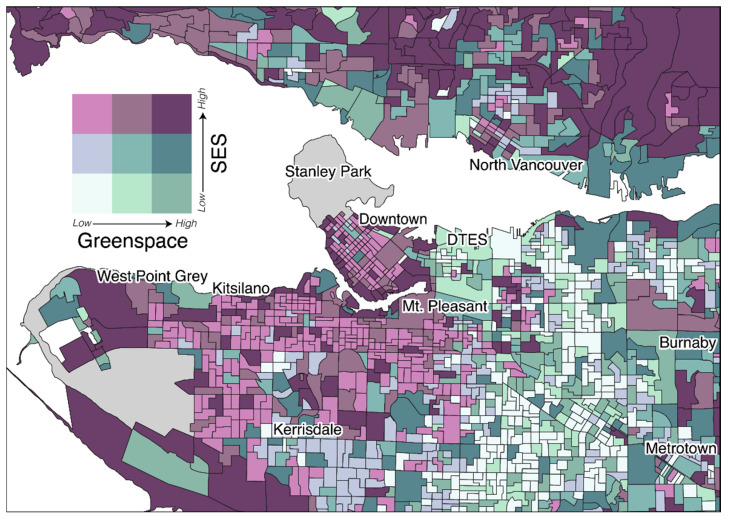
Bivariate map illustrating the spatial relationship between neighborhood SES and green/blue space. The amount of green/blue space within a DA determined the area’s score. A tertile of VANDIX scores informed the SES in a DA. Regions shaded in gray did not have SES data since they are federal and provincial lands.

The distribution of Strava runs were 36.9% for women and 59.4% for men along the 99,094 unique segments. Individuals between 35 and 54 years of age accounted for most of the users running on segments (43.3%), followed by individuals between 20 and 34 (32.7%), 55 and 64 (6.5%), those 65 years and older (0.9%), and those between 13 and 19 years of age (0.4%). These demographic distributions are not representative of the Metro Vancouver population [69] indicating a bias in the Strava data toward men and those between 35 and 54 years of age. Approximately 32.8% of the route segments were contiguous with green or blue spaces. There was also a clear spatial relationship between neighborhood SES and green/blue space (Figure A1). Running activities were concentrated in areas with higher combined SES and green/blue space, as well as in DAs with high relative SES, such as Downtown Vancouver and Kitsilano (Figure 2). In general, higher volumes of running were observed in parks and along water bodies. 

### 3.2. Generalized Linear Model Results

The results of the GLM revealed Strava running activities were 3.81 times more likely (IRR = 3.81, *p* < 0.001) to occur in areas contiguous to green and/or blue spaces (Table 2). Runners were 21% less likely (IRR = 0.79, *p* < 0.001) to run on segments in DAs characterized by low relative urbanicity compared to areas with moderate urbanicity. Runners were slightly more likely to choose routes in DAs with moderately low (IRR = 1.16, *p* < 0.001) and moderately high (IRR = 1.2, *p* < 0.001) urbanicity than in moderately urban DAs. Additionally, runners were 2.06 times more likely to log runs in highly urbanized DAs than in moderately urban DAs (IRR = 2.06, *p* < 0.001). Running was 49% less likely (IRR = 0.51, *p* < 0.001) to occur in DAs with the lowest SES compared to areas with moderate SES. Running was 16% less likely to occur in areas with moderately low SES (IRR = 0.84, *p* < 0.001) while areas with moderately high SES were 37% more likely (IRR = 1.37, *p* < 0.001). Running was 2.26 times more likely in DAs with high SES compared to moderate SES DAs (IRR = 2.26, *p* < 0.001). 

Neighborhood preferences were generally consistent across gender and age categories; however, the influence of each predictor varied between groups. Table 3 compares the influence of neighborhood characteristics on runner volume for men and women. The largest gender discrepancy was observed in the influence of green/blue space. Women were 3.44 times more likely (IRR = 3.44, *p* < 0.001) to run on segments contiguous with green/blue space while men were 3.2 times more likely (IRR = 3.2, *p* < 0.001). Table 4 shows the GLM results for the five age categories. For all ages, the likelihood of runner increased as relative neighborhood SES and urbanicity increased. Green/blue space was most influential for runners 65 years and older (IRR = 4.77, *p* < 0.001), followed by runners between 13 and 19 years old (IRR = 4.66, *p* < 0.001).

## 4. Discussion

As Strava’s popularity increases, analysis of crowdsourced data has growing potential to inform planning for active communities [70]. This study assesses the influence of neighborhood characteristics on running decisions and demonstrates the utility of user-generated data in determining conducive running environments. Findings illustrate the impacts of the urban environment on running patterns and the influence of age and gender on route preferences. Urbanized neighborhoods characterized by high SES and the presence of green/blue space significantly promoted running across ages and genders. Adolescents, older adults, and women were most influenced by the presence of green/blue space, which increased running likelihood among these populations. The results can inform planning for active communities and better understand runner preferences to identify incentives and remove resistance to running in low participation areas. 

Green and blue space significantly increased the odds of running along a route for all groups in our study. A growing amount of literature reports a similar relationship, concluding that green space increases the likelihood of physical activity which improves the wellbeing of urban communities [24,25,71,72,73]. Similarly, blue space is associated with increased running and has been linked to higher satisfaction within a running environment [27,30,31,74,75]. Green and blue space had a significant positive association on running popularity even though less than 33% of Strava segments were contiguous with these environments. This indicates that natural areas are sought out by Strava runners. Whether parks constitute the entire route or are a fixture of a longer run is unknown, due to the aggregated structure of the data, and likely depends on individual proximity to green space. The highest run volumes were found in areas consisting of green and blue space such as Stanley Park (a Vancouver park approximately 25% larger than Central Park) which includes a 10 km Seawall path). The observed behaviors of our study population align with the green space preferences of runners identified in previous surveys which emphasized tree-covered spaces and perceived greenness [1,32].

This study found there was generally a greater likelihood of running as urbanicity increased, except for moderately low urbanicity areas which had slightly more running than moderately urban areas. Possibly, this reflects a greater number of green/blue spaces in moderately low urban areas than in moderate urban areas. The concurrent influence of urbanicity and green space on route popularity could be explained by the presence of micro green spaces (i.e., pocket parks, community gardens) and tree-lined streets in population dense areas. Sidewalks and tree-lined streets, which are preferred characteristics identified in runner surveys [1,3,32], are scattered throughout Downtown Vancouver. Further, Downtown Vancouver and surrounding areas are composed of grid-like streets with high connectivity, which makes route creation and navigation easier for runners. However, high population density most likely explains the concentration of runs in the downtown core. The population increases significantly during work hours, meaning that a number of suburban commuters run downtown on breaks and before or after work. This means that runs may occur near a person’s work rather than the neighborhood they live in.

The lower volume of Strava runners in certain areas with high population density possibly reflects a less hospitable environment for runners. If a neighborhood does not have features conducive to running, then residents are less likely to engage with the activity there. Urban design can promote exercise through effective land use planning and widely available public transport [76,77]. Crowdsourced data from Strava can inform infrastructure improvements for municipalities interested in increasing their runnability. However, features that promote running, such as maintained paths or streetlights, often reflect the SES of a community. In our study, it is unknown whether a runner travels to a running site or if they run in their own neighborhood. Persons with high SES likely have greater transport access and can travel further to run in more conducive environments. At the same time, runners with high SES likely live in areas with more attractive running environments and may not have to leave their neighborhood to run. Runs recorded on Strava were concentrated in high SES neighborhoods or green spaces that often require a personal vehicle to access efficiently, meaning low SES may be a significant barrier to running in Vancouver.

This study found routes in areas with higher relative SES increased the odds of running among Strava users. SES mediates running in both urban and rural areas, and has traditionally been measured at the household level. Low household SES has been related to lower levels of exercise in general, which places disadvantaged individuals at greater risk of developing health problems associated with sedentary lifestyles [64,65,78]. Studies have linked lower physical activity to the inaccessibility of appropriate fitness settings as a result of SES and proximity; however, running has not been evaluated explicitly [78]. Running is more accessible than other forms of fitness that require equipment, participation costs, or specified settings; however, socio-economic barriers to running engagement remain. Low SES significantly decreased the likelihood of running in a neighborhood. Strava data over-represent more socio-economically privileged groups, which partially explains less representation of activities in lower SES areas [79]. It is also likely that Strava users are avoiding low SES neighborhoods. The Downtown Eastside (DTES) in Vancouver has a concentrated homeless population and subsequently the lowest relative SES in the study area. Adjacent to the DTES, Downtown Vancouver constitutes a large portion of the highest SES neighborhoods. Despite their proximity, there are noticeably fewer Strava runs recorded in the DTES. Part of the reason behind this discrepancy may be due to the quality and frequency of parks in a neighborhood. Studies have found that spatial access to green space follows patterns of SES, and often those with low SES have less access to adequate green space [80,81]. In Vancouver, the inequitable distribution and management of green space is documented [82,83]. 92% of the Vancouver population lives within a five minute walk from greenspace; however the quality and unequal distribution of these spaces is apparent [82].

Findings differed only slightly by gender but suggest that women were less likely to exercise in low SES areas and DAs with lower urbanicity compared to men. The largest difference occurred for green/blue space, which was associated with running for women significantly more than men. Green space proximity is linked to increased likelihood of exercise, particularly for women and young adults [84], and increased wellbeing specifically for youth and older adults in an urban setting [38]. In our study, adolescent and older adult runners were influenced by green space significantly more than all other ages. The two groups were also the most underrepresented by the data. The lack of representation may be linked to less access to or interest in Strava. Alternatively, this may reflect a lack of engagement in running for fitness among adolescents and older adults. Considering the positive influence of green space on these runner groups, strategic development of outdoor spaces in low engagement areas may promote running among younger and older populations.

This study is limited in several ways. The categorization of green/blue space using land use data does not capture the range of settings people consider green space, such as streets lined with trees or residential gardens. Additionally, the 15 m buffer around green/blue spaces, while highly accurate, may be ineffective in situations where a house or highway stands between the space and a running route. Further, data are objective and therefore do not reflect the perceptions of runners while they navigate a route, including their perceived access to a space and the quality and safety of an area for running. Since this is a preliminary study demonstrating the utility of Strava data, many factors were not used in the analysis including variables that likely influence runnability significantly.

Other limitations were related to the Strava dataset. Strava data give researchers the capacity to understand runner behaviors in depth and contribute to running-friendly planning policies. However, the structure of user data is beyond researchers’ control and deeper analysis is hindered by the need to protect individual privacy. For privacy protection, user activity was binned into counts of five and spatially aggregated to segments which prevented a more detailed analysis. The data were temporally aggregated to the entire year thus limiting the ability to draw conclusions about daily and seasonal variation among users, which likely is a significant factor in running. The categorization of age and gender also presents challenges. The age categories produced by Strava are not classified by equal intervals, rather data are grouped into dramatically different age ranges (i.e., 35 to 54, 13 to 19). The categories of men, women, and unspecified do not acknowledge the diversity of genders and limit the ability to analyze gender diverse Strava users. Gender and age were unable to be simultaneously measured in the models since runner data consists of counts for age groups and counts by gender which do not overlap. Lastly, Strava data are not representative of the general population [79] and findings may only reflect a small proportion of runners in Metro Vancouver. Strava is utilized by those with access to the technology, which excludes people without smart devices. Strava users are often younger men who tend to have higher SES, and individuals with low SES are likely underrepresented [47,79,85]. Reaching underrepresented populations would allow for a more accurate account of the routes selected by runners from all backgrounds. Further, results reflect the unique conditions of a coastal metropolitan area in the Pacific Northwest. However, we feel the overall patterns of the study population can inform planning for conducive running environments if paired with qualitative data explaining the reasoning behind runner preferences. 

## 5. Conclusions

The purpose of this study is to determine the association between neighborhood characteristics and running, while demonstrating the utility of crowdsourced Strava data for understanding the influence of the environment on runner behavior. The potential of Strava data is beginning to be explored for urban planning through cyclist studies [40]. Our preliminary research uses crowdsourced data to provide insight on the under-explored preferences of runners. The results provide a deeper understanding of environmental correlates with popular running routes and explain differences by age and gender. High neighborhood SES, the presence of green and/or blue space, and high population density were associated with increased running for all ages and genders. Age and gender altered the degree of influence environmental factors had on the likelihood of running, warranting further age and gender-based analyses. Findings can be used to promote participation in running. The high volume and fine spatial resolution of Strava data make for a comprehensive account of how urban spaces are navigated by runners. Future work should consider efforts to make crowdsourced fitness data more inclusive and consider Strava’s impact on communities through qualitative approaches. Future directions of this research will compare neighborhood preferences between cyclists and runners, and further elaborate on the potential of Strava data to inform urban planning for active communities. 

## Figures and Tables

**Table 1:** Neighborhood Variables.

Variable	Description	Source	Measure
Green and/or Blue Space	Recreation areas, open spaces, protected areas, waterbodies	Metro Vancouver 2016 Land Use	Contiguity (0/1)
SES	VANDIX values for DAs	2016 Census	Quintiles
Urbanicity	Population density for DAs	2016 Census	Quintiles

**Table 2:** Overall Running Activities.

Variable	IRR	CI
Green/Blue Space	3.81 *	3.73–3.90
Relative Urbanicity		
Low	0.79 *	0.77–0.82
Moderately Low	1.16 *	1.13–1.20
Moderate	Referent	
Moderately High	1.2 *	1.17–1.24
High	2.06 *	1.99–2.12
VANDIX (SES)		
Low	0.51 *	0.49–0.52
Moderately Low	0.84 *	0.81–0.86
Moderate	Referent	
Moderately High	1.37 *	1.33–1.41
High	2.26 *	2.19–2.33

Referent is the attribute that serves as the point of comparison for interpretation of the results. * *p*-value < 0.001.

**Table 3:** Strava Runners by Gender.

	Men		Women	
Variable	IRR	CI	IRR	CI
Green/Blue Space	3.2 *	3.13–3.27	3.44 *	3.36–3.53
Relative Urbanicity				
Low	1.01	0.98–1.04	0.92 *	0.89–0.96
Moderately Low	1.2 *	1.17–1.24	1.21 *	1.17–1.26
Moderate	Referent		Referent	
Moderately High	1.24 *	1.20–1.27	1.29 *	1.25–1.33
High	2.36 *	2.29–2.43	2.5 *	2.41–2.59
VANDIX (SES)				
Low	0.56 *	0.54–0.57	0.52 *	0.50–0.54
Moderately Low	0.81 *	0.79–0.83	0.81 *	0.79–0.84
Moderate	Referent		Referent	
Moderately High	1.4 *	1.36–1.44	1.47 *	1.42–1.52
High	2.58 *	2.51–2.66	2.69 *	2.60–2.79

* *p*-value < 0.001.

**Table 4:** Strava Runners by Age Group.

	13 to 19		20 to 34		35 to 54		55 to 64		65 and Older	
Variable	IRR	CI	IRR	CI	IRR	CI	IRR	CI	IRR	CI
Green/ Blue Space	4.66 *	3.13–3.27	3.34 *	3.25–3.43	3.3 *	3.23–3.38	3.28 *	3.18–3.39	4.77 *	4.49–5.06
Urbanicity										
Low	0.84 *	0.98–1.04	1.13 *	1.09–1.18	0.89 *	0.87–0.92	0.98	0.93–1.02	0.76 *	0.70–0.83
Mod Low	0.7 *	1.17–1.24	1.32 *	1.28–1.37	1.15 *	1.12–1.19	1.2 *	1.15–1.26	1.1	1.01–1.19
Mod	Ref		Ref		Ref		Ref		Ref	
Mod High	0.73 *	1.20–1.27	1.35 *	1.31–1.40	1.21 *	1.18–1.25	1.28 *	1.23–1.34	0.86 *	0.79–0.93
High	0.98	2.29–2.43	2.95 *	2.84–3.06	2.25 *	2.18–2.32	1.99 *	1.90–2.08	1.51 *	1.39–1.64
SES										
Low	0.63 *	0.54–0.57	0.54 *	0.52–0.56	0.55 *	0.54–0.57	0.47 *	0.45–0.49	0.38 *	0.35–0.41
Mod Low	1.02	0.79–0.83	0.79 *	0.76–0.82	0.83 *	0.80–0.85	0.8 *	0.76–0.83	0.64 *	0.59–0.69
Mod	Ref		Ref		Ref		Ref		Ref	
Mod High	1.27 *	1.36–1.44	1.56 *	1.50–1.62	1.38 *	1.34–1.43	1.26 *	1.20–1.31	1.35 *	1.25–1.47
High	1.84 *	2.51–2.66	2.75 *	2.65–2.85	2.63 *	2.55–2.72	2.28 *	2.18–2.38	2.55 *	2.34–2.77

* *p*-value < 0.001.

## Data Availability

The data presented in this study are available on request from the corresponding author. The data are not publicly available due to privacy restrictions.
